# Heat Transfer Coefficients Analysis in a Helical Double-Pipe Evaporator: Nusselt Number Correlations through Artificial Neural Networks

**DOI:** 10.3390/e21070689

**Published:** 2019-07-14

**Authors:** Arianna Parrales, José Alfredo Hernández-Pérez, Oliver Flores, Horacio Hernandez, José Francisco Gómez-Aguilar, Ricardo Escobar-Jiménez, Armando Huicochea

**Affiliations:** 1 CONACyT—Centro de Investigación en Ingeniería y Ciencias Aplicadas (CIICAp), Universidad Autónoma del Estado de Morelos, Cuernavaca C.P. 62209, Mexico; 2 Centro de Investigación en Ingeniería y Ciencias Aplicadas (CIICAp), Universidad Autónoma del Estado de Morelos, Cuernavaca C.P. 62209, Mexico; 3 CONACyT—Tecnológico Nacional de México/CENIDET, Interior Internado Palmira S/N, Col. Palmira, C.P. Cuernavaca 62490, Mexico; 4 Tecnológico Nacional de México/CENIDET, Interior Internado Palmira S/N, Col. Palmira, C.P. Cuernavaca 62490, Mexico

**Keywords:** heat transfer coefficients, artificial neural network, Nusselt number, helical heat exchangers

## Abstract

In this study, two empirical correlations of the Nusselt number, based on two artificial neural networks (ANN), were developed to determine the heat transfer coefficients for each section of a vertical helical double-pipe evaporator with water as the working fluid. Each ANN was obtained using an experimental database of 1109 values obtained from an evaporator coupled to an absorption heat transformer with energy recycling. The Nusselt number in the annular section was estimated based on the modified Wilson plot method solved by an ANN. This model included the Reynolds and Prandtl numbers as input variables and three neurons in their hidden layer. The Nusselt number in the inner section was estimated based on the Rohsenow equation, solved by an ANN. This ANN model included the numbers of the Prandtl and Jackob liquids as input variables and one neuron in their hidden layer. The coefficients of determination were $R^{2}>0.99$ for both models. Both ANN models satisfied the dimensionless condition of the Nusselt number. The Levenberg–Marquardt algorithm was chosen to determine the optimum values of the weights and biases. The transfer functions used for the learning process were the hyperbolic tangent sigmoid in the hidden layer and the linear function in the output layer. The Nusselt numbers, determined by the ANNs, proved adequate to predict the values of the heat transfer coefficients of a vertical helical double-pipe evaporator that considered biphasic flow with an accuracy of ±0.2 for the annular Nusselt and ±4 for the inner Nusselt.

## 1. Introduction

In a wide variety of industrial processes, the main purposes of heat exchange devices are to transform and generate energy. The design and sizing of any heat exchanger require the calculation of the heat transfer coefficients, which quantitatively indicate the heat transfer carried out inside the device [1]. Theoretical and experimental investigations have been focused on understanding and representing this energy exchange from empirical equations supported by experimental data. Each of these expressions is governed by specific restrictions, such as geometric parameters and flow conditions, which are generally considered as a single phase.

Generally, in the case of heat exchangers with helical pipelines, it is known that the flow behavior has a greater complexity compared to exchangers that include straight pipes [2]. Due to the generation of vortices, induced by the interaction between the viscous forces and the centrifugal force, a disturbance in the flow is generated, which increases the rate of the heat transfer and the friction losses—a behavior referred to as secondary flow [3].

If a helical exchanger transports a biphasic flow, specifically gas-liquid, the complexity is even higher due to the sudden changes in the flow pattern. These phenomena are mainly caused by the sliding between the phases, the orientation of the pipe, and the void fraction, among other additional variables and the relationships between them [4]. Therefore, to know and understand these phenomena, it is necessary to estimate the values of the heat transfer coefficient in the helical exchangers that contain biphasic flows, and hence any method that leads to their calculation is considered useful.

One of the methods commonly used to estimate heat transfer coefficients is the Wilson plot method, proposed by Wilson in 1915 and subsequently modified in 1957 [5]. This traditional method quantifies the heat flow for each section by the exchanger through the measurement of the global temperature between the two fluids involved and their rate of heat transfer [6]. One of the most significant advantages of this technique is that it avoids the complex direct measurement of the surface temperature and thereby decreases experimentation costs. The method requires that the fluid in one section of the exchanger be kept at a constant rate and temperature, whereas in the other heat exchanger section, the fluid rate and temperature vary. In [7], a method modification was made to improve its accuracy. The method considers both temperature and fluid rates as dynamic variables in both heat exchanger sections. This modification requires partial knowledge about one of the thermal resistances of the exchanger and an iterative scheme to obtain the unknowable thermal resistance [8].

In general, if the fluid is considered as single-phase, the Nusselt number is a function of some properties of the fluid as well as geometrical and flow parameters. However, for a two-phase flow, other considerations are necessary due to the inclusion of the characteristics of the gas–liquid interface such as surface tension, latent heat of vaporization, and pressure [9].

Consequently, if a helical double-pipe exchanger considers two different flow types, there are hence two convective thermal resistances that need their respective Nusselt number models. However, due to the complexity of the phenomenon, the formulation of the models requires the use of non-linear optimization tools that provide adequate values.

Regarding this concept, one of the proposed methods with more application is artificial neural networks (ANNs), due to their remarkable ability to solve complex problems with high accuracy [10]. According to Mohanraj et al. [11], ANNs can infer non-linear parameters involved in heat transfer processes with minimal errors since they are based on experimental data and therefore do not require any specific analytical equation.

Several investigations have coupled the use of neural networks for the description of heat transfer phenomena. For a thermoacoustic heat exchanger, Rahman and Zhang [12] proposed an approximate model to predict the heat transfer coefficient with high accuracy and an error rate of 3.2%. Azizi and Ahmadloo [13] developed a model to predict the heat transfer coefficient during the condensation of R134a in a smooth inclined tube from 440 experimental points, obtaining a coefficient of determination of 0.995.

Concerning the prediction of the Nusselt number, Ghritlahre and Prasad [14] developed a neural model for a solar air heater, obtaining a coefficient of determination of 0.997. Esfe [15] modeled the Nusselt number and the pressure drop of an Ag/water nanofluid in a double tube heat exchanger, reaching coefficients of determination greater than 0.995.

In particular, studies on the estimation of heat transfer coefficients in helical exchangers are few [16]. The different techniques used in many investigations have been focused in single-phase flows where flow variations on one side of the exchanger were kept constant to simplify the problem [17,18]. On the other hand, studies that involve phase changes have been based on the application of single-phase correlations due to the complex and expensive local measurements in this type of exchanger when coupled in a particular system. Therefore, alternative methodologies for their study with two-phase flows can help the design and increase the efficiency of the helical exchangers as required.

Hence, to estimate the convective heat transfer coefficients in a vertical helical double-pipe evaporator with accuracy, the aim of this research was to develop the Nusselt number correlations to accurately describe the energy exchange in each section of the helical evaporator by applying the modified Wilson plot method and artificial neural networks. It is important to mention that an experimental database (1109 values) from a vertical helical double-pipe evaporator coupled to an absorption thermal transformer with energy recycling (AHTER) and integrated into a water purification system was obtained. Consequently, the main contributions of the research are the following: (1) an accurate estimation of the Nusselt number for the annular section of a vertical helical double-pipe evaporator based on the modified Wilson plot method and solved by applying artificial neural networks; the Nu depends on the dimensionless numbers of the liquid Reynolds ($Re_{l}$) and the liquid Prandtl ($Pr_{l}$); (2) an accurate estimation of the Nusselt number for the inner section of a vertical helical double-pipe evaporator based on the Rohsenow equation, which depends on the dimensionless numbers of liquid Prandlt ($Pr_{l}$) and Jackob (Ja), which are the principal parameters that characterize the biphasic behavior.

## 2. Experimental Setup

In this section, the experimental system, which consists of a vertical helical double-pipe evaporator coupled to an absorption heat transformer with energy recycling integrated into a water purification system, is described. The absorption heat transformer with energy recycling AHTER is considered as an energy saving device because its main function is to raise the temperature of a residual heat source and recycle it into another process [19]. Several studies on the absorption heat transformer were developed to find solutions to reduce fossil fuel usage, as well as to mitigate environmental pollution. Particularly, the CIICAp-UAEM installed an AHTER where the delivered useful heat was supplied for a water purification process by simple distillation.

**Figure 1:**
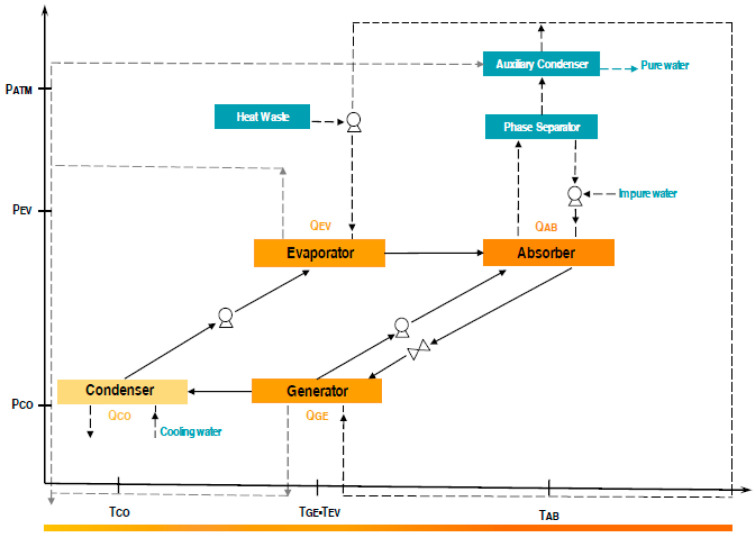
Schematic diagram of the absorption heat transformer with energy recycling integrated into the water purification system.

The evaporator is one of the main components of the AHTER in addition to the absorber, the generator, and the condenser [20]. (Figure 1) shows the closed thermodynamic cycle of AHTER, in which the heat source with a low energy level was transferred to the generator and the evaporator, while in the absorber, a high-level heat source was generated. Once the interaction finished between the components, which operated under two different pressures, the cycle was restarted.

To operate the system, it was necessary to simulate the waste energy source through an electrical resistance controlled by a voltage regulator. The flow rate of the heating water was measured with a flow meter with an operating range of 1.5 to 15 $\frac{l}{\min}$. The temperatures were measured using T-type thermocouples calibrated with a reference thermometer +0.1 °C. The measured pressure range was 101.35 to 103.42 kPa. The data recording time was 10  s for each test from the Agilent Technologies (Mexico City, México) 34970A instrument and a 34901A multiplexer module with 20 voltage input channels. The specifications of the instrumentation are presented in (Table 1).

As part of the experimental study, the variables of operation of the main components in motionless condition were determined. An experimental database (1109 values) from a vertical helical double-pipe evaporator coupled to an absorption thermal transformer with energy recycling (AHTER) and integrated into a water purification system was obtained.

The performance of the helical evaporator was a critical factor in maximizing the coefficient of performance (COP) of the system. During the operation of the absorption heat transformer, tests were conducted at different pressures, obtaining different temperatures to find the optimal conditions and thereby increase the efficiency of the system. The average experimental conditions of the evaporator are presented in (Table 2).

**Figure 2:**
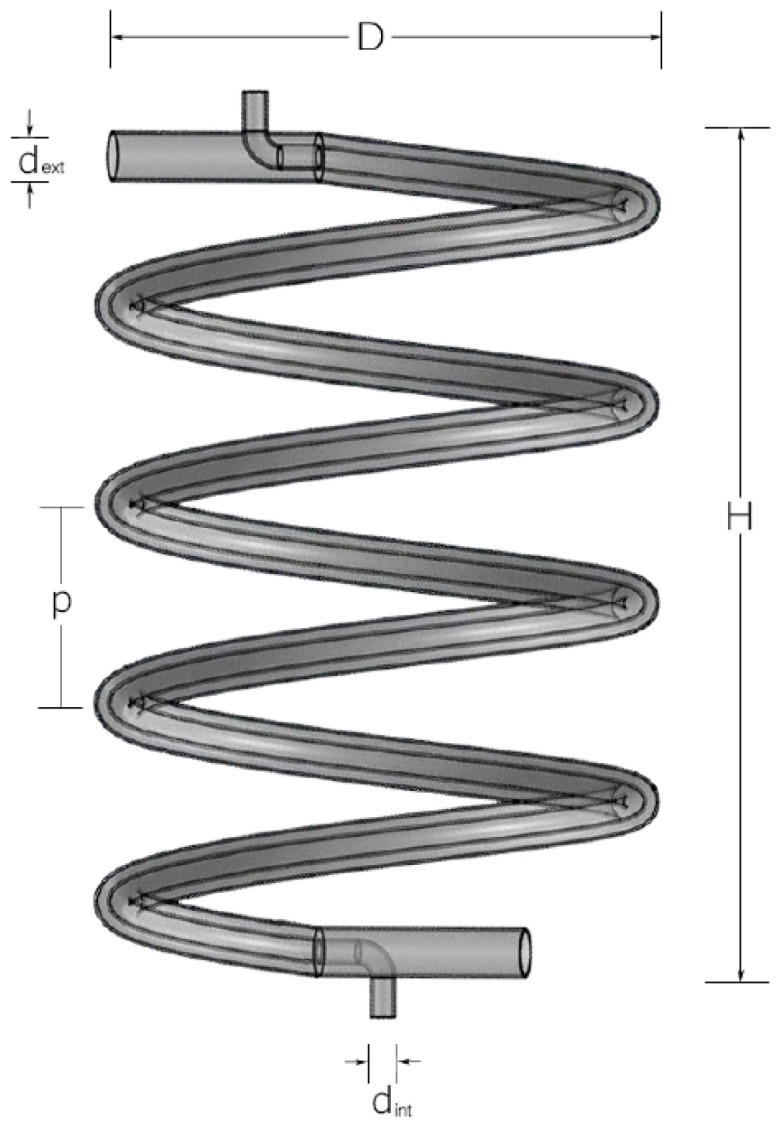
Schematic diagram of an experimental helical double-pipe evaporator.

In particular, the evaporator is a stainless-steel heat exchanger with concentric helical pipes where two sections are recognized: the inner section and the annular section. The working fluid with which the evaporator operated was water in a countercurrent flow. In order to minimize the heat exchanger heat loss to the environment, the helical evaporator was insulated by 19 mm of flexible thermal insulation. A geometrical scheme of the vertical helical double-pipe evaporator is shown in (Figure 2) and the geometric parameters are shown in (Table 3).

## 3. ANN Modified Wilson Plot Methodology

The methodology used to obtain the values of the heat transfer coefficients for each section of the helical evaporator—the result of the modified Wilson plot method coupled with artificial neural networks—is described below.

In the helical evaporator, the temperatures of the two fluids varied along the pipe, derived from the heat exchange and their heat transfer flux, which can be calculated from:

(1) $$\dot{Q}=\dot{m}\Delta H$$

where $\dot{m}$ is the mass flow and ΔH represents the amount of exchanged energy.

Likewise, as expressed in the following equation, the heat flux is a function of the global heat transfer coefficient U, the temperature difference ΔT and the transfer surface A, which can apply to both the inner section and the annular section. It is important to mention that for this type of exchanger, the appropriate temperature difference was considered as $\Delta T_{log}$.

(2) $$\dot{Q}=UA_{a}\Delta T_{log}$$

(3) $$\Delta T_{log}=\frac{\left(T_{a\;outlet}-T_{i\;inlet}\right)-\left(T_{a\;inlet}-T_{i\;outlet}\right)}{\ln\left(\frac{\left(T_{a\;outlet}-T_{i\;inlet}\right)}{\left(T_{a\;inlet}-T_{i\;outlet}\right)}\right)}$$

**Figure 3:**
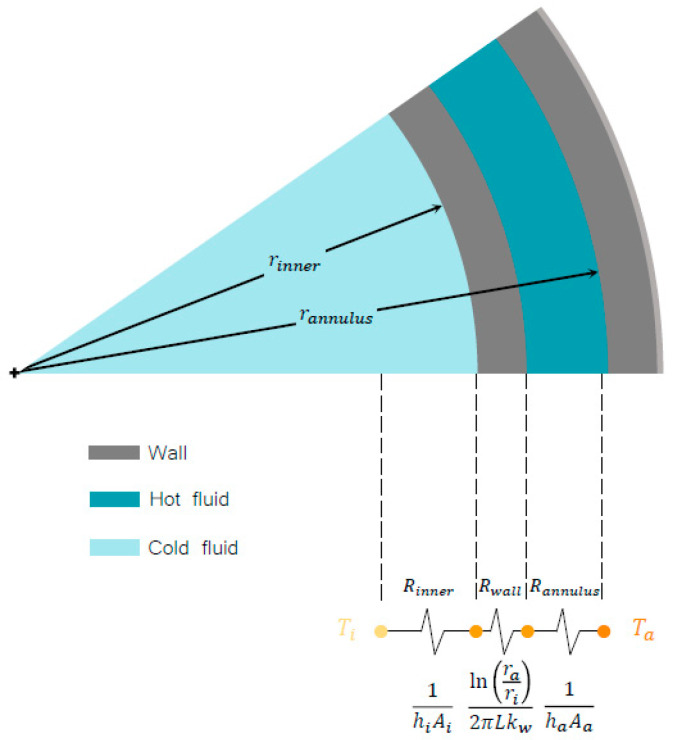
Thermal resistance in the helical double-pipe evaporator.

Following the original work of Wilson, it was considered convenient to adopt the concept of a thermal circuit. As shown in (Figure 3), for the helical evaporator, three types of resistances were considered: the resistance of the fluid circulating through the inner pipe, the resistance of the fluid circulating through the annular pipe, and the conductive resistance of the pipe wall. In this case, to simplify the problem, the resistances by incrustation were disregarded.

Therefore, the global thermal resistance was represented as the sum of the individual resistances, as shown in the following equation:

(4) $$\frac{1}{UA}=R_{i}+R_{w}+R_{a}$$

The wall resistance $R_{w}$ is independent of the fluids and is a function of the geometric parameters and the thermal conductivity of the pipe, so it can be considered constant in all the tests. It is worth mentioning that the wall resistance was lower compared to the sum of the other resistances.

On the other hand, the inner pipe resistance $R_{i}$ and the annular pipe resistance $R_{a}$ are commonly expressed in terms of the convective heat transfer coefficient h with respect to the respective surface. Therefore, by replacing the values of each resistance, the equation can be rewritten as:

(5) $$\frac{1}{UA}=\frac{1}{h_{i}A_{i}}+\frac{\ln\left(\frac{r_{o}}{r_{i}}\right)}{2\pi k_{w}L}+\frac{1}{h_{a}A_{a}}$$

According to the above modification, the unknown thermal resistance can be inferred from the quantification of the known individual resistances and the overall thermal resistance.

Specifically, concerning the convective thermal resistance of the internal fluid, it is known that during the exchange, the working fluid underwent a phase change as a consequence of the vaporization process. When this process occurs, the calculation of the heat transfer coefficient can be considered average, as it is assumed to be independent of the quality. In general, the main variable that controls the mechanism of bubble formation is the excess temperature, that is, the difference in temperature that exists between the temperature of the fluid and the corresponding saturation temperature, as expressed below:

(6) $$\Delta T_{x}=T_{f}-T_{sat}$$

If $\Delta T_{x}$ varies within the range of 5 to 30 °C, which is presented in this case, it is considered as nucleated boiling, characterized by the complex prediction of the bubble formation on the surface. To correlate the experimental data in the nucleate boiling regime, the equation proposed by Rohsenow [21] was used, expressed as:

(7) $$h_{i}=\frac{\mu_{l}h_{fg}{\left[\frac{g\left(\rho_{l}-\rho_{v}\right)}{\sigma }\right]}^{\frac{1}{2}}{\left[\frac{Cp_{l}\Delta T_{x}}{C_{sf}h_{fg}Pr}\right]}^{3}}{\Delta T_{x}}$$

Following the previous correlation, it can be observed that boiling depends mainly on the latent heat of the vaporization of the fluid and the surface tension in the liquid–vapor interface, in addition to some properties of the fluid in each phase. The main advantage of the Rohsenow correlation is that, at any pressure and heat flow, the particular fluid–surface relationship $C_{sf}$ can be known; in particular, for a water–stainless steel combination, it has a value of 0.013 [22].

After quantifying the previous resistance, it was possible to deduce the value of the annular convective heat transfer coefficient $h_{a}$. However, because the fluid in the annular section did not suffer a phase change, the values obtained can be expressed in terms of the Nusselt number, as seen in the following expression:

(8) $$Nu_{a}=\frac{h_{a}D_{eq}}{k_{l}}$$

The physical experience indicates that the Nusselt number depends greatly on the Reynolds and Prandtl numbers since the Reynolds number is a measure of the movement of mixing associated with the flow, and the Prandtl number characterizes the relationship between the properties of viscosity and thermal conductivity of the fluid. Therefore, the obtained data can be correlated employing a relation of the type:

(9) $$Nu_{a}=f\left(Re_{l},{\mathrm{Pr}}_{l}\right)$$

However, according to the non-linear behavior of the data obtained, the use of the artificial neural network (ANN) tool was necessary. The ANN, inspired by brain structure and functionality, is a non-linear method characterized by adaptive learning and the high ability to represent associations accurately from a quantity of information [23].

The ANN model can express the complex relationship between the most important variables of a determinate process through adequate neural architecture, which mainly consists of interconnected processing units of denominated neurons. Generating a neural model requires the organization of the neurons in three layers: the input layer, the hidden layer, and the output layer. The input layer considers the variables of the phenomenon to be modeled, and the output layer includes the variables to be predicted. The hidden layer refers to the number of neurons needed to describe the complexity of the phenomenon, that is, the number of unknown connections that exist between the input variables concerning the output variables.

It is possible to find an adequate architecture from the variation of the number of neurons in the hidden layer with the help of an optimization algorithm, an activation function, and a certain number of iterations [24]. The most suitable optimization algorithm is the Levenberg–Marquardt backpropagation algorithm (LMA), since it allows the decrease of the mean square error (MSE), as opposed to other backpropagation algorithms [25].

In regard to the activation functions, the use of the hyperbolic tangent transfer function in the hidden layer and the linear function in the output layer was established, as recommended by Karlik and Olgac [26]. This model is represented through the following equation:

(10) $$Output_{k}=\overset{N}{\underset{n=1}{\sum }}\left[Wo_{\left(k,n\right)}\left(\frac{2}{1+e^{\left(-2\sum_{r=1}^{R}\left(Wi_{\left(n,r\right)}P_{\left(r\right)}+b_{1}{}_{\left(n\right)}\right)\right)}}-1\right)\right]+b_{2}{}_{k}$$

where N is the number of neurons in the hidden layer, *W_i_* is the weights in the input hidden layer, *b*_1_ is the number of the bias in the hidden layer, *W_o_* is the weights in the hidden output layer, *R* is the number of neurons in the input layer, *k* is the output neuron number, and *b*_2_ is the number of the bias in the output layer.

Additionally, it was recommended to perform a sensitivity analysis to determine the influence of the variables on the neural model of the study phenomenon through the Garson equation, which is based on the neural network weight matrix obtained, as shown in the equation below:

(11) $$I_{j}=\frac{\sum_{m=1}^{m=Nh}\left(\left(\frac{\left|W_{jm}^{ih}\right|}{\sum_{k=1}^{Ni}\left|W_{km}^{ih}\right|}\right)\left(\left|W_{mn}^{ho}\right|\right)\right)}{\sum_{k=1}^{k=Ni}\left\{\left(\sum_{m=1}^{m=Nh}\left(\left(\frac{\left|W_{jm}^{ih}\right|}{\sum_{k=1}^{Ni}\left|W_{km}^{ih}\right|}\right)\left(\left|W_{mn}^{ho}\right|\right)\right)\right)\left(\left|W_{mn}^{ho}\right|\right)\right\}}$$

where $I_{j}$ is the relative importance of the $j^{th}$ input variable on the output variable, and $N_{i}$ and $N_{h}$ are the number of neurons in the input and hidden layers, respectively. The superscripts i, h, and o represent the input, hidden, and output layers, respectively. The subscripts k, m, n are the input, hidden, and output neurons, respectively [27].

## 4. Results and Discussion

In this section, the following main results are presented: (4.1) the development of the annular Nusselt number model, inferred from the modified Wilson plot method with ANN; (4.2) the development of the inner Nusselt model based on the Rohsenow correlation by ANN; (4.3) the evaluation of both models with the experimental data in the helical evaporator.

### 4.1. Annular Nusselt Number Correlation

In the first instance, and according to the modified Wilson plot method, to obtain the set of the values of the annular heat transfer coefficient, it was necessary to calculate the values of the known resistances for 1109 data obtained from the experimental tests, which were: the global thermal resistance, the wall resistance, and the convective resistance of the internal fluid.

The values obtained from the annular convective heat transfer coefficient can be expressed in terms of the Nusselt number. Therefore, an artificial neural architecture was used to generate a suitable model capable of predicting the Nusselt number values for the annular section of the helical evaporator.

**Figure 4:**
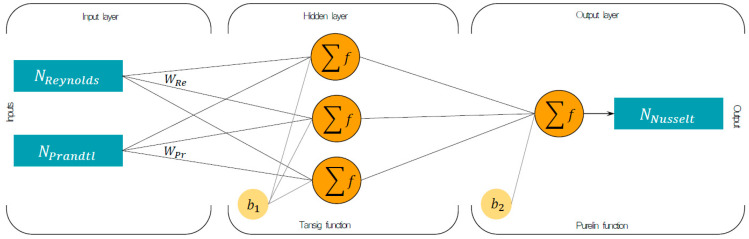
The architecture of the neural model of the annular Nusselt number with two inputs.

Due to the dimensionless nature of the Nusselt number, the numbers of the liquid Reynolds ($Re_{l}$) and the liquid Prandtl ($Pr_{l}$) were considered as dimensionless input variables due to their importance as critical parameters that adequately describe the heat transfer for single-phase flows. Hence, the neural architecture, which used the hyperbolic tangent transfer function and linear transfer function to predict the annular Nusselt number, is shown in (Figure 4), where the weights $W_{Re_{l}}$ and $W_{Pr_{l}}$ represent the synaptic neural connections.

The ANN toolbox of Matlab® software (R2015b, Mathworks®, Natick, MA, USA) was used to develop the annular Nusselt number with the ANN architecture. To train the model, the input variables were normalized between 0 and 1, obtained dividing the value with the maximum value of each variable. This dataset was divided into three blocks, 70% of which was assigned to training, while the remaining 30% was used for the validation (15%) and testing (15%) processes in equal proportions.

During the ANN training and according to the Levenberg–Marquardt backpropagation algorithm, the different numbers of the neurons in the hidden layer, where the weights and biases iteratively adjust to reduce the deviation of the predicted values of the network, were evaluated. The numbers of the neurons were optimized by the analysis of the statistical residuals between the output $Nu_{a\;ANN}$ and the target values $Nu_{a\;exp}$, following the equation:

(12) $$RMSE=\sqrt{\frac{1}{r}\overset{r}{\underset{i=1}{\sum }}{\left(Nu_{a\;\exp\left(i\right)}-Nu_{a\;ANN\left(i\right)}\right)}^{2}}$$

Avoiding the over-fitting, the optimal number of neurons in the hidden layer was three neurons with a coefficient of determination of 0.9973. (Table 4) shows the nine weights and four biases that were selected and replaced in Equation (10) of the ANN architecture. By simplifying the expression, the equation for predicting the annular Nusselt number values obtained was:

(13) $$Nu_{a}=\frac{13563}{1997\left(1+e^{a}\right)}+\frac{4131}{482\left(1+e^{b}\right)}-\frac{776}{353\left(1+e^{c}\right)}-\frac{1487}{1225}$$

where

$$a=\frac{73}{3035}Re_{l}-\frac{15759}{154}Pr_{l}-\frac{9122}{131}$$

$$b=-\frac{37}{4641}Re_{l}+\frac{7775}{262}Pr_{l}+\frac{2833}{96}$$

$$c=-\frac{196}{2195}Re_{l}+\frac{23880}{53}Pr_{l}-\frac{7415}{148}$$

**Figure 5:**
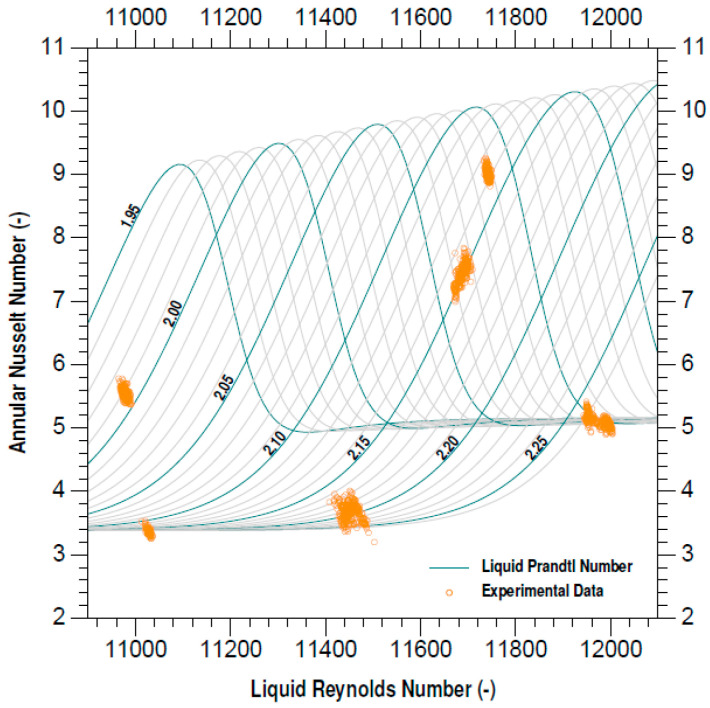
Annular Nusselt number plot.

Based on the new model in the experimental pressure range, which can be observed in (Figure 5), the Nusselt number is susceptible to the minimum variation of the Prandtl number since the pressure on the fluid plays an important role in the variation of the characteristics of the fluid and thence on the Nusselt number.

### 4.2. Inner Nusselt Number Correlation

To simplify the correlation of Rohsenow, an alternative ANN model for the inner section of the evaporator was proposed. Since the evaporation depends on the characteristics of the vapor–liquid interface and the properties of the fluid in each phase, it was necessary to propose two dimensionless numbers that describe these parameters adequately.

In nucleate boiling, the energy absorbed during phase change is mainly related to the motion of the liquid that flows around the bubbles that are formed during the process. This behavior is typified by the excess temperature $\Delta T_{x}$, the latent heat of vaporization $h_{fg}$, and the liquid heat capacity $Cp_{l}$. The dimensionless number that considers the aforementioned variables is the Jackob number, which was therefore proposed as an essential variable to describe the inner Nusselt number.

(14) $$Ja=\frac{Cp_{l}\Delta T_{x}}{h_{fg}}$$

Another important dimensionless parameter considered was the liquid Prandtl number for its importance in heat transfer in single-phase flows. This parameter was evaluated at the saturation temperature corresponding to the local pressure in the fluid. Therefore, in the new ANN model proposed, the input variables considered were the dimensionless numbers of the liquids Prandtl and Jackob.

The training procedure of the ANN model for the internal section was the same as that with which the previous model was obtained. The optimal number of neurons in the hidden layer was one neuron with a coefficient of determination of 0.9998. (Table 5) shows the values of the weights and biases obtained for the second model of the Nusselt number. These values were replaced in Equation (10) and the inner Nusselt number can be defined by the following equation:

(15) $$Nu_{i}=\frac{40097}{14\left(1+e^{\left(\frac{673}{3883}Pr_{l}-\frac{5945}{302}Ja+\frac{1954}{1125}\right)}\right)}-\frac{22231}{65}$$

**Figure 6:**
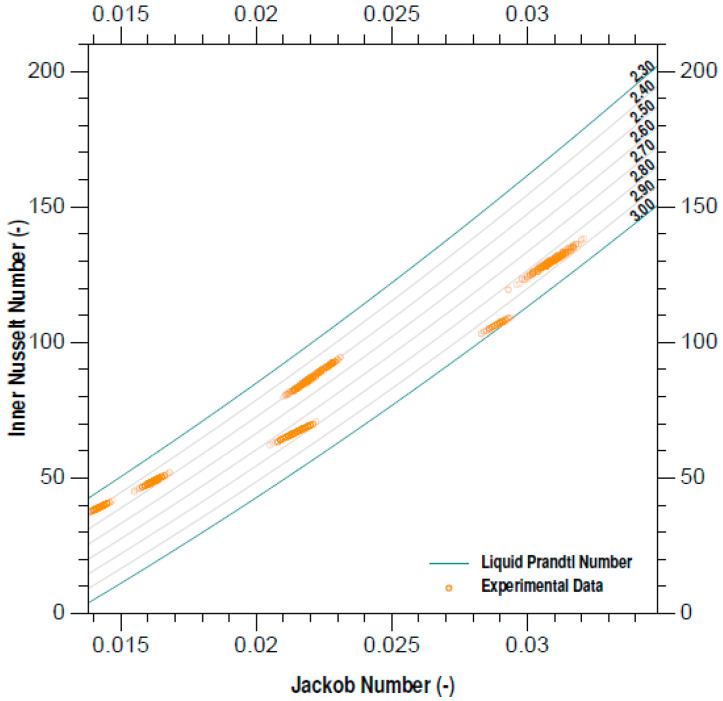
Inner Nusselt number plot.

Analogously, the development of the new equation in the experimental pressure range suggests that it is sufficient to describe the inner Nusselt number from the numbers of the liquids Prandtl and Jakob, as can be seen in (Figure 6). As expected, the changes in the liquid properties, related to the experimental pressure, generated significant changes in the values of the Prandtl number.

### 4.3. Evaluation of Both Models

**Figure 7:**
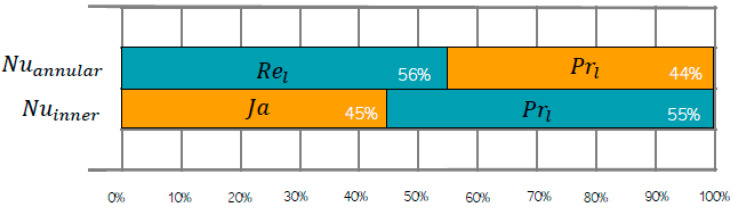
Sensitive analysis of Nusselt number models inferred from the ANN.

When carrying out a sensitive analysis of the models proposed, according to the equation of Garson, the relative importance of the Reynolds number can be corroborated at 56% and the Prandtl number at 44% on the annular Nusselt number. Additionally, the number of the liquid Prandtl showed a 45% importance, while the number of Jakob, 55% of the inner Nusselt number, as can be seen in (Figure 7).

**Figure 8:**
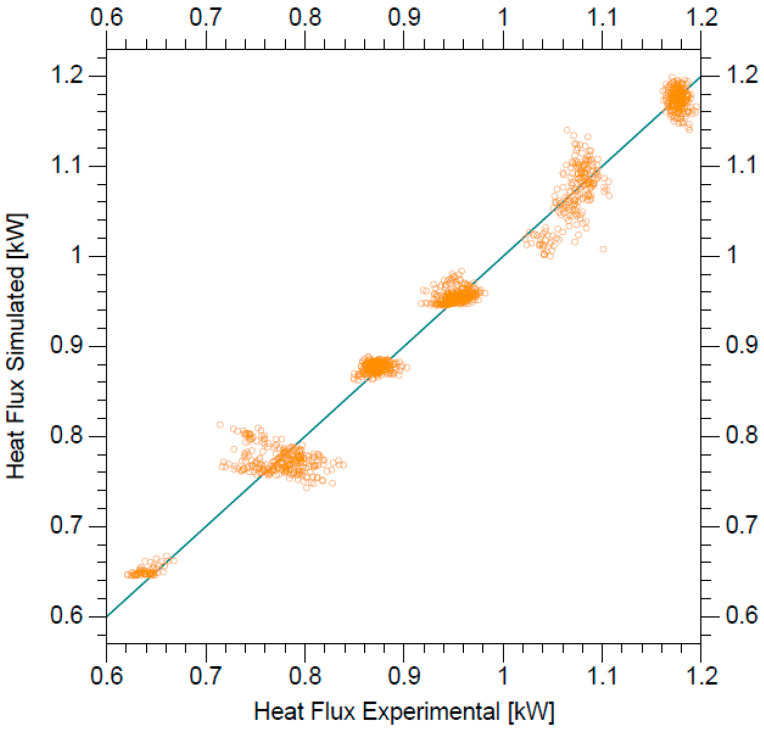
Comparison of the experimental and simulated heat flux.

Figure 8 shows the experimental and simulated heat flux for all the test, where the suitable ability for the prediction of heat flux with the two new empirical Nusselt models can be visualized.

## 5. Conclusions

In this research, two new empirical correlations of the Nusselt number, estimated by ANN, were proposed to determine the heat transfer coefficients of a vertical helical double-pipe evaporator. To train the ANNs, an experimental database of 1109 values was obtained from a vertical helical double-pipe evaporator coupled to an absorption heat transformer with energy recycling integrated into the water purification system.

The Nusselt number calculation in the annular section of the evaporator was based on the modified Wilson plot method solved by ANN. The best architecture for the development of the model was obtained with three neurons in the hidden layer and the dimensionless numbers of the liquid Reynolds and the liquid Prandtl were considered. In the inner section of the evaporator, the Nusselt number calculated was based on the Rohsenow equation solved by an artificial neural network model where the dimensions of the Prandtl and Jackob liquids were considered as input variables.

It was possible to corroborate that the Nusselt numbers obtained by the ANN were satisfactory to predict the values of the heat transfer coefficients and hence their suitable capacity for quantifying heat transfer in a vertical helical double-pipe evaporator by considering biphasic flow.

## Figures and Tables

**Table 1:** Specification of the instruments of the setup.

	Specifications	Accuracy
Voltage Regulator	Variac Trademark Powerstar with 240/120 V 40–280 V, 10 A	-
Flowmeter	Parker FBZ-44 1.5 to 15 L/min	±3% full scale
Thermocouples	Type T	±0.2 °C
Pressure transducer	Cole-Parmer	±0.25% full scale

**Table 2:** Average experimental conditions of the experimental helical double-pipe evaporator.

Test	Pressure [kPa]	$T_{i_{inlet}}$ [°C]	$T_{i_{outlet}}$ [°C]	$T_{a_{inlet}}$ [°C]	$T_{a_{outlet}}$ [°C]
**1**	21.40≤P≤22.00	30.99	64.37	79.49	76.44
**2**	23.13≤P≤24.43	27.66	66.34	82.61	78.93
**3**	25.35≤P≤26.40	32.36	67.38	79.67	75.55
**4**	34.15≤P≤35.55	26.58	75.40	86.71	82.32
**5**	36.22≤P≤37.17	28.22	77.26	83.95	78.87
**6**	38.56≤P≤39.48	28.65	78.12	85.86	80.50

**Table 3:** Dimensions of the helical double-pipe evaporator.

	Internal Pipe (mm)	Annular Pipe (mm)
**External diameter**	9.52	19.05
**Internal diameter**	6.22	15.75
**Helical diameter**	240	240
**Laps**	4	4
**Length**	3500	3500
**Height**	300	300

**Table 4:** The weight values and biases for the ANN annular Nusselt number.

	Values		
**Weights**	**IW**		**LW**
	99.697355	−146.65890	−0.37335047
	2.1571707	−5.9141821	0.4.6977472
	−566.97914	494.99369	0.11868204
**Bias**	**B1**	**B2**	
	36.069390	0.58024316	
	34.188246		
	26.478381		

**Table 5:** The weight values and biases for the ANN inner Nusselt number.

	Values		
**Weights**	**IW**		**LW**
	−0.25622385	0.31595107	10.360415
**Bias**	**B1**	**B2**	
	−0.86844425	7.8860045	

## Nomenclature

A	Area $\left(m^{2}\right)$	T	Temperature (°C)
AHTER	Absorption heat transformer with energy recycling	U	Overall heat transfer coefficient $\left(\frac{W}{m^{2}{}^{\circ}C}\right)$
ANN	Artificial neural network	W_i_, W_o_	Matrix of weights
b_1_, b_2_	Matrix of bias	$\Delta T_{x}$	Excess temperature (°C)
Cp	Heat capacity ($\frac{J}{kg\;K}$)	**Greek Letters**	
$C_{sf}$	Fluid-surface constant (−)	ρ	Density $\left(\frac{kg}{m^{3}}\right)$
D	Helical diameter (m)	σ	Superficial tensión $\left(\frac{N}{m}\right)$
$D_{eq}$	Equivalent Diameter (m)	μ	Dynamic viscosity (Pa s)
d	Internal diameter (m)	k	Thermal conductivity $\left(\frac{W}{m\;{}^{\circ}C}\right)$
g	Acceleration due to gravity $\left(\frac{m}{s^{2}}\right)$		
H	Enthalpy $\left(\frac{J}{kg}\right)$	**Subscript**	
h.	Heat transfer coefficient $\left(\frac{W}{m^{2}\;{}^{\circ}C}\right)$	a	Annular
$h_{fg}$	Latent heat of vaporization ($\frac{J}{kg}$)	exp	Experimental
L	Length (m)	f	Fluid
$\dot{m}$	Mass flow rate $\left(\frac{kg}{s}\right)$	g	Vapor
Nu	Nusselt number (−)	i	Internal
Pr	Prandtl number (−)	l	Liquid
p	Pitch (m)	sat	Saturation
$\dot{Q}$	Heat flux (W)	sim	Simulation
Re	Reynolds number (−)	w	Wall
RMSE	Root mean square error		
